# Characterization of the complete chloroplast genome of the *Camellia nitidissima*, an endangered and medicinally important tree species endemic to Southwest China

**DOI:** 10.1080/23802359.2018.1501304

**Published:** 2018-08-27

**Authors:** Meng-Meng Liu, Ze-Peng Cao, Jun Zhang, Da-Wei Zhang, Xiao-Wei Huo, Gang Zhang

**Affiliations:** a College of Traditional Chinese Medicine, Hebei University, Baoding, China;; b College of Pharmaceutical Science, Key Laboratory of Pharmaceutical Quality Control of Hebei Province, College of Pharmaceutical Science, Hebei University, Baoding, China;; c College of Pharmacy and Shaanxi Provincial Key Laboratory for Chinese Medicine Basis & New Drugs Research, Shaanxi University of Chinese Medicine, Xi’an, China;; d Institute of Bioinformatics and Medical Engineering, School of Electrical and Information Engineering, Jiangsu University of Technology, Changzhou, China

**Keywords:** *Camellia nitidissima*, chloroplast genome, high-throughput sequencing, endangered species

## Abstract

The *Camellia nitidissima* is an endangered tree species native to Southwest China with high economic and medicinal values. Genetic information of *C. nitidissima* would provide good knowledge for the conservation of this wild resource. In this article, we characterized the complete chloroplast genome of *C. nitidissima* using Illumina sequencing technology. The size of circular genome is 157,247 bp, containing a large single copy (LSC) region of 86,880 bp and a small single copy (SSC) region of 18,258 bp. The LSC region and SSC region are separated by a pair of inverted repeat regions (IRa and IRb), each of 26,068 bp. In total, 136 genes are encoded in this cp genome, including 89 protein-coding genes (81 species), 39 tRNA genes (30 species), and 8 rRNA genes (4 species). The overall G + C content of the chloroplast genome is 37.3%. The phylogenetic analysis suggests that *C. nitidissima* is closely related to *C. petelotti*.

## Introduction

*Camellia nitidissima* C.W. Chi is an evergreen shrub that belongs to the family Theaceae, distributed naturally in Southwest China, and mainly found in the Guangxi Zhuang Autonomous Region of China (He et al. 2018). It was first reported in the Chinese medical classics Ben Cao Gang Mu 400 years ago and has been widely used to treat hepatitis with jaundice, hypertension, diarrhea, and irregular menstruation (He et al. 2015). As a result, Because of the lack of effective protection and the increasing commercial demand, the wild resources of *C. nitidissima* has undergone a large population reduction (Zhou et al. 2017).

It was reported that the genetic variation in wildlife populations plays an important role in the survival and adaptive evolution of populations in a dynamic environment (Dlugosch and Parker 2008). However, the gene structure and genomic information of the *C. nitidissima* was still unknown. In this study, we report the complete chloroplast (cp) genome sequence of *C. nitidissima* using Illumina sequencing technology.

The *C. nitidissima* plant sample, in our study, was collected from Guangxi Medicinal Botanical Garden. Total genomic DNA was extracted from 0.2 g fresh leaves of *C. nitidissima* using the RN44 DNA plant mini kit (Aidlab, Beijing, China) following the manufacturer’s instructions. A total amount of 2 μg high-quality DNA was used for sequencing with the Illumina Miseq. The chloroplast genome was assembled using MITObim version 1.8 (Hahn et al. 2013). The cpDNA sequence of *Camellia huana* (KY626040) (Wang et al. 2017) was set as a reference. The DOGMA online tool (Wyman et al. 2004) was used to annotate the cp genome of *C. nitidissima*. We have deposited the annotated cp genomes into GenBank and got the accession number MH382827.

The cp genome length of *C. nitidissima* is determined to be 157,247 bp and a GC content of 37.3%. The cp genome included a pair of inverted repeats (IRs, 26,068 bp each other) that divide the whole sequence into two single-copy regions, namely large single-copy region (LSC; 86,880 bp), and small single-copy region (SSC; 18,258 bp). Among this cp genome, 136 functional genes are predicted. Among the 136 encoded genes in the *C. nitidissima* cp genome, 113 are unique genes (including 80 protein-coding genes, 29 transfer RNA genes, and four ribosomal RNA genes) and 18 are duplicated genes in the IR regions. The 18 duplicated genes include eight protein-coding gene, 10 tRNA genes, and four rRNA genes. Nineteen distinct genes contain one intron and another two genes (*ycf3*, *clpP*) possess a couple of introns.

**Figure 1.**
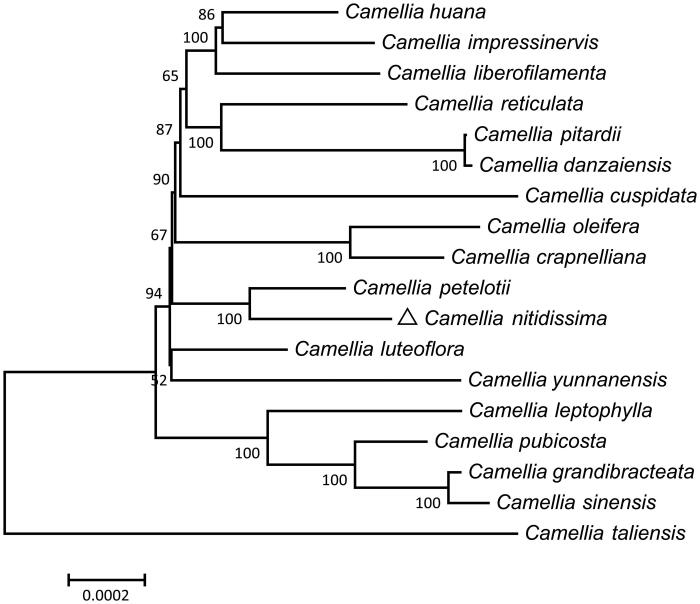
Phylogenetic relationships among 18 *Camellia* species based on the neighbor–joining (NJ) analysis of 77 cp PCGs. The bootstrap values next to the branches are based on 500 resampling.

MEGA7 (Kumar et al. 2016) was used to carry out the phylogenetic analysis based on the 18 aligned sequences with the neighbor–joining (NJ) method (Figure 1). The phylogenetic analysis clarified that *C. nitidissima* is closely related to its congeneric *C. petelotti.* The result was similar to a previous study by Vijayan et al. (2009) suggesting that *C. nitidissima* and *C. petelotti* were close relatives. The complete *C. nitidissima* cp genome sequence would be available for the genetic diversity studies of *Camellia* and show a new perspective for understanding the conservation and restoration of *C. nitidissima*.
